# Acceptability and feasibility of an evaluation table to assess the competency of general medicine interns during ambulatory rotations in Brest

**DOI:** 10.1186/s12909-024-05357-7

**Published:** 2024-06-06

**Authors:** Brieux Longépé, Audrey Madec, Jérôme Fonseca, Lucas Beurton-Couraud, Marie Barais, Delphine Le Goff

**Affiliations:** 1 Department of General Practice, University of Western Brittany, 22, av. Camille Desmoulins, 29238 Brest, FR France; 2 ER 7479 SPURBO, University of Western Brittany, 22, av. Camille Desmoulins, 29238 Brest, FR France

**Keywords:** Competency-based education, Internship and residency, General practice, Teaching, Educational assessment

## Abstract

**Background:**

General practitioner interns need to acquire the expected clinical, communication, personal and professional competencies. Internship evaluations use qualitative evaluation tables to assess competency acquisition. However, there is no standardised evaluation table used in France. Some faculties use the exhaustive, precise, and manageable Exceler evaluation tool. We aim to evaluate opinions of General practice interns in Brest about the acceptability and feasibility of using the Exceler evaluation tool to monitor competency acquisition during internships.

**Methods:**

This qualitative study used intern focus groups. Six-open ended questions with optional follow-up questions were asked. Cards from the Dixit® game were used to guide and facilitate discussion. Open, axial, then integrative analysis of the verbatim was performed.

**Results:**

This is the first study to evaluate intern opinions about GP internship evaluations using focus groups. Participants felt that the quality of existing evaluations was insufficient, and it was difficult to monitor their progress. Adapting evaluations to individual profiles and backgrounds seemed necessary. Exceler appeared to be a possible solution due to its content validity, flexibility of use and accessibility. However, there were comments about possible modifications.

**Conclusions:**

Analysing opinions of tutors, supervisors and other practice centers could help identify potential barriers and reveal solutions to facilitate its implementation and use.

**Trial registration:**

Not applicable.

**Supplementary Information:**

The online version contains supplementary material available at 10.1186/s12909-024-05357-7.

## Background

In France, the General Practitioner (GP) diploma was reformed in 2004 marking a pedagogical turning point for the specialty [1].

Health professionals make their decisions through their ability to activate cognitive processes and knowledge within the context of clinical reasoning. This ability to reason forms the core of medical professional competence [2–5]. French GP interns are certified as being competent once they have acquired the expected clinical, communication, personal and professional competencies [6–8]. Competency encompasses theoretical knowledge, including illness and patient history, and practical knowledge, including clinical examination, performing consultations and communication. Ambulatory rotations are one pedagogical method among many others to acquire these competencies. Their organisation, similar to companionship, is based on the competency-based learning model which is inspired by the constructivist methods [9]. It follows the learning paradigm, emphasising the interns’ active role in learning and the purpose of that learning [10–12]. Theoretical teaching alone, such as magistral lessons or books, does not guarantee the correct assimilation of GP competencies. Practical teaching is also required to acquire and use these competencies in various situations [13].

Internship evaluations to assess competency acquisition can be either summative where the intern’s learning is compared to a benchmark at the end of a course or rotation, or formative where student learning is monitored, providing ongoing feedback to staff and interns [9, 10]. Competencies are practical objects complex to evaluate in reel-life situations. Tools to explain the conceptional framework of their acquisition, record and guide interns progression are essential. Thus, evaluations should be based on valid, reproducible, and feasible descriptive qualitative evaluation tables which illustrate specific learning situations such as the Calgary-Cambridge table [14]. These enable a more concrete and reproducible evaluation but are harder to design and use. The results from these evaluation tables can be interpreted using either criterion-referenced interpretation which seeks to measure the intern’s level of learning according to predetermined objectives, or a normative interpretation which compares the intern to a group or reference but is less focused on the learning achieved [15]. These two interpretations can be complementary depending on the desired objective.

To date, there is no standardised evaluation table used in France for intern evaluations. Currently in Brest, a table with a purely summative evaluation function is used. This is detrimental since formative evaluations have been shown to benefit intern performance and confidence [16]. However, some faculties use a computerised evaluation table, called “Exceler” in this study, which was developed by the University of Limoges [17]. This qualitative and criterion-referenced tool has a hierarchy of indicators used for each competency and allows several evaluations to be superimposed. It can be used by interns and supervisors and enables both formative and summative evaluations. Its exhaustiveness, precision and manageability give it validity and reproducibility but potentially reduce feasibility. Exceler could be a useful tool for monitoring competency acquisition and providing clarity about the final phase of intern training thus helping interns perfect their future practice (Appendix 1).

This study aims to evaluate opinions of general medicine interns in Brest about the acceptability and feasibility of using the Exceler evaluation tool to monitor intern competency acquisition during internships.

## Method

### Study design

This qualitative study used focus groups to explore GP intern opinions about the Exceler evaluation tool.

### Participants

General medicine interns in Brest, France, were recruited through a post on the intern Facebook group. Additional interns were contacted directly using a snowball approach, for example through the Facebook messenger application or during in-person meetings. Recruitment was spread over three semesters on two separate faculty sites.

### Data collection

Focus groups were chosen to collect the data since they enhance dynamic exchange between participants. Each focus group included four to five participants. BL, a male general medicine intern, who received qualitative research training based on the French reference book for qualitative studies [18] and guided by his supervisor, conducted all the focus groups. One focus group was conducted at Quimper boarding school, the others were at BL’s home. The researcher knew some participants prior to the focus groups but had no prior relationship with others. At the start of the focus groups, BL explained his interest in the research topic and the participants received information about the purpose of the research and their right to correction and withdrawal. Each participant provided informed consent. The Exceler tool was only presented during the focus groups, so the participants were not influenced prior to the discussion. Six open-ended questions were used with follow-up questions if necessary (Appendix 2). To guide and facilitate discussion during the focus groups, cards with dreamlike illustrations from the Dixit® game were used. Six different Dixit® cards were randomly given to each participant. For each question, the participant had to choose the card which best represented their answer to the question. For example, ‘Which card best represents your opinion about the Exceler tool?’. They placed this card face down on the table in front of them. As well as choosing a card, the participant had to write down one or more keywords or sentences relating to the card. The selected cards were then shuffled with a few additional cards and laid out for all participants to see. Each participant then shared their keywords one by one, the researcher noted them on a virtual whiteboard visible to all so that the others could try to guess which card was whose. Participants then discussed the keywords to come up with a concise answer to the question. Field notes were not made during the focus groups. Each focus group was audio recorded, fully transcribed and anonymised. Transcripts were sent to the participants for correction. Only participants and BL were present during the focus groups.

### Data analysis

**Fig. 1:**
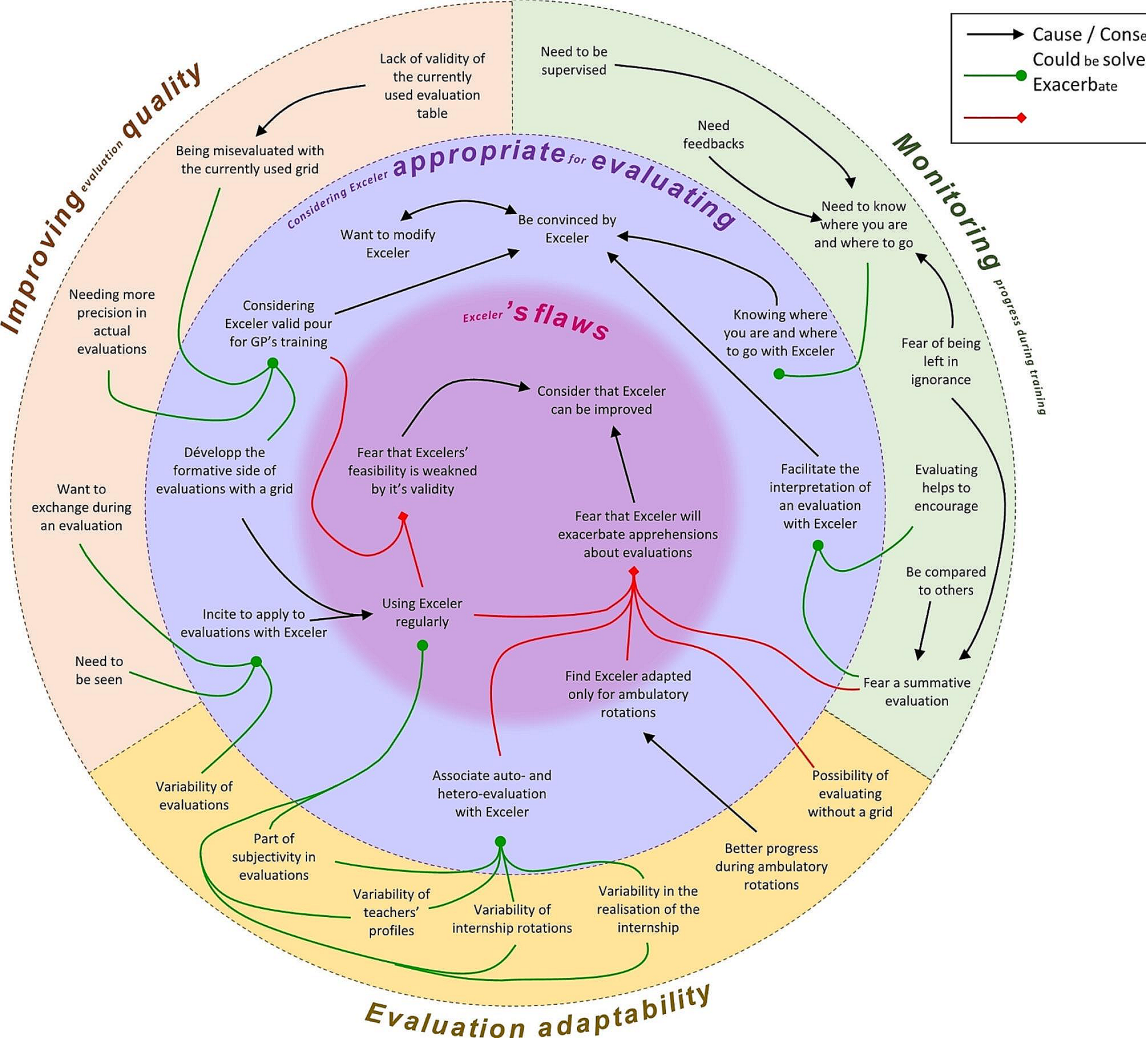
Mind map created from verbatim analysis

Open, axial then integrative analysis of the verbatim was performed. Two researchers separately performed two initial blinded analyses to ensure triangulation. The interpretation process of verbatims was synthesized through a coding tree. From initial labelling, several properties were created through discussion. A third researcher could be consulted if a consensus wasn’t reached. Properties were finally categorised using an inductive approach. As verbatims were coded one after another, each one could bring new elements which required iterative coding, especially for the firsts focus groups. A mind map, a schematic representation of the participants’ thoughts, was created from the coding tree (Fig. 1). QSR NVivo11® software was used for data collection and analysis. Participants were given the opportunity to provide feedback on the results.

### Ethics statement

The authors confirm that all methods were carried out in accordance with the Declaration of Helsinki. This study was approved by Brest primary care ethics committee (n° 29SP23.002).

## Results

### Focus group and participant characteristics

Five focus groups each including four to five people were conducted between August 2021 and March 2022. The interviews lasted between 138 min and 195 min. Data saturation was obtained once any new element was revealed during the coding process. This theoretically means that every different opinion about de subject was collected, which occurred at the fifth interview.

A total of 18 interns participated in the focus groups. They were mostly women (61%, *n* = 11) with an average age of 27 years. Three participants were in their second autonomous ambulatory rotation, the others had completed or were in their first ambulatory rotation. None of the participants had completed the gynaecology/paediatrics ambulatory rotation. Participant characteristics are shown in Table 1.

**Table 1:** Characteristics of interns participating in the focus groups

	Age	Gender (Male/Female)	Current semester	Number of ambulatory rotations completed/ongoing	Number of semesters in the 1st ambulatory rotation	Number of semesters in the autonomous 2nd ambulatory rotation
P01	27	M	4	1	3	/
P02	27	M	4	1	4	/
P03	28	M	4	1	4	/
P04	27	M	4	1	3	/
P05	27	F	4	1	3	/
P06	26	F	4	1	3	/
P07	27	F	4	1	3	/
P08	26	F	4	1	3	/
P09	26	F	4	1	3	/
P10	27	F	4	1	4	/
P15	25	F	3	1	2	/
P16	32	M	5	1	3	/
P17	27	F	5	1	3	/
P18	26	F	5	1	4	/
P19	27	F	5	2	3	5
P20	28	M	5	2	4	5
P21	26	M	3	1	1	/
P22	27	F	5	2	3	5

### Focus group analysis

Analysis revealed five main categories:

### Improving evaluation quality

When discussing the current evaluation system, participants felt that the discussion and exchange elements were the most important; *“an evaluation is done orally, it’s an exchange”* (P1). It gave them the opportunity to be listened to and considered: *“It’s the principle of the evaluations, to overcome the difficulties”* (P3).

Most participants felt the currently used evaluation tool was poor. It was described as being *“imprecise”, “unsuitable”* or *“infantilising”.* Some stated that *“some themes don’t correspond at all to what we do”* (P6), other felt that *“the letters used to grade us are completely useless, it’s not at all precise to put and A, B or C”* (P19). Participants also reported that their supervisors *“say the same thing when completing the tables”* (P6).

Some felt the current system was *“sloppy”* and was done *“hastily”* (P9). Others felt that only comments made in the general observations section “*had any relevance”* (P2).

Several participants would have liked the evaluation tool to be adapted to each of their internship rotations; *“We all have to follow the same model which is quite clear. We all do emergencies, general medicine and paediatrics or gynaecology, each with their own objectives. The tables should be adapted to these objectives”* (P8).

### Monitoring progress during training

Most participants expressed difficulty following their progress with the current evaluation system; *“Typically during the first course you’re given an A for everything, even though there is room for improvement”* (P3). Furthermore, the lack of benchmarks could create a certain fear or frustration about evaluations; *“I am always frightened of what they’ll say”* (P5), *“Sometimes you are only reproached at the end, when, in fact, if you had done a mid-term evaluation it could have been improved before the end of the course”* (P17). Others felt the stress of comparison with other students; *“Did you only get a B?”* (P8). However, many participants felt that evaluations were encouraging and gave them confidence; *“She was reassuring so it went well”* (P18), “*They don’t want to break you”* (P1).

The ideal evaluator was described as someone who had been able to monitor their progress by observing them work, such as their rotation supervisor; *“They were doctors who we had seen throughout the semester. They had also taken feedback from other doctors whom we had been with”* (P22). However, many participants had concerns about their internship tutor evaluating their competencies since they never observed them working in practice. Interns felt that their internship tutor was *“unable to assess my skills”* (P8) because *“we never see him, he doesn’t know us at all”* (P20).

### Evaluation adaptability

Participants highlighted variations in evaluation methods and evaluators; *“We don’t have meetings with our tutors”* (P19), *“Some tutors take it really seriously, others just can’t be bothered”* (P15). Initially, standardising evaluations using an evaluation tool seemed a good solution. However, being evaluated using qualitative criteria appeared *“hard”* as there is always *“a level of subjectivity”.* Others felt it was dangerous to want to standardise competency acquisition; *“training is not reproducible so putting us all in the same box is very difficult”* (P17). It is therefore necessary to adapt the evaluation to each individual; *“Everyone is different, some interns are better at communication than others”* (P19).

Some participants felt that an evaluation tool was not essential; “What matters most is the overall evaluation of the course, not completing tables” (P1), “We had a meeting with the tutors, there was no evaluation tool, just feedback” (P18).

### Considering Exceler as appropriate for evaluating

Most participants were positive about the Exceler tool, and all participants agreed that it could help to improve evaluations. They appreciated the precision of the indicators, their validity regarding training and the improved objectivity; *“It’s a big improvement compared to what we have, it’s much more suitable, it’s better”* (P8), *“It’s more objective”* (P7).

Being able to target their *“strengths and weaknesses”* to guide and adapt their training seemed to be an added value; *“You can see the things you need to improve. You can question yourself more easily about things you need to work on”* (P2). Furthermore, the evaluation interpretation appeared clearer due to the summary page; *“It’s visual, it’s easier to understand your level compared with grades like A, B, C”* (P10). Participants also felt the Exceler tool could be complementary to other evaluation methods; *“There are other tables for training, but this is for evaluating your practice”* (P8). Others even considered how to modify it to make it more appropriate; *“Instead of yes/no, you could have a scale from 0-100%”* (P15).

The possibility of using the tool for self-evaluation and/or evaluation with the tutor was discussed in each focus group. Some felt it was interesting to use the tool for one or the other, but all participants agreed that both evaluations were necessary for its optimal use; “*It would be best to fill in the tables with the doctor you are training with. There are things that are easier for them to complete and others which are easier for self-assessment”* (P4). Some participants felt that the exhaustiveness of the Exceler tool would help evaluations; *“It forces you to complete it correctly, you have to have a real discussion about your internship”* (P04).

### Exceler’s flaws

Most participants felt that the tool would only be useful if it was used regularly; *“Once or twice a year would be good”* (P15). They also wanted it to be used just for formative evaluations and not summative; *“It should just be a support tool”* (P21).

Many found it unfortunate that the Exceler tool could not be applied to hospital internships; *“It doesn’t evaluate our entire internship. Hospital rotations are at least half of our course”* (P3). Others felt it would only be useful for the ambulatory rotations.

Some participants were put off by the exhaustiveness of the Exceler tool; *“There are a lot of things on the table, it’s a little scary. You have to validate so many things, it almost seems academic”* (P18). Furthermore, almost all participants felt that the Exceler could be improved. Some indicators seemed less relevant, were repeated, and could appear illogical; *“For the most part there is good progression but there are elements which aren’t logical”* (P8). In addition, one participant found the digital version difficult to use; *“I need the paper version”* (P7).

A mind map was created showing the interactions and relationships between the results (Fig. 1).

## Discussion

To our knowledge, this is the first French study to evaluate intern opinions about GP internship evaluations using focus groups. Participants felt that the quality of content of existing evaluations was insufficient, and it was difficult to monitor their progress. Furthermore, the lack of progression benchmarks could be a source of anxiety. Adapting evaluations to individual profiles and backgrounds seemed necessary. Exceler appeared to be a possible solution due to its content validity, flexibility of use and accessibility. However, there were comments about possible modifications.

The negative feedback about the existing evaluation tool which is currently used in Brest were expected and mainly related to its weak formative role. The indicators in the existing tool are not designed for general medicine competencies so there is weak validity [5]. Furthermore, using a Likert scale can produce significant variability and offer little information about progression [19, 20]. However, it has good feasibility. It can be assumed that this quality was deliberately prioritised since it is only used for summative evaluations.

In France, several medical faculties have chosen to use criterion-referenced evaluation tables [21–24] due to the lack of validity of existing tables, variability of evaluations, insufficient self-evaluation, and lack of follow-up and progress benchmarks. The criterion-referenced approach allows intern performance levels to be assessed on a descriptive scale using multiple measures established from authentic situations [25]. Of the evaluation tools found in the literature, Exceler is the most successful in terms of validity and reproducibility. It has been designed to standardise evaluations and has been tested and revised several times [17]. Its regular use during medical training is part of the competency-based learning model.

Competency is defined as a “a complex knowledge of what to do relying on effectively harnessing and combining a variety of internal and external resources within a family of situations” [26, 27] and this definition is the core of competency-based learning. The competency-based learning model is based on the learning paradigm through active participation of the intern, promoting competency acquisition, and increasing the feeling of personal effectiveness and recognition as a true professional. Competency-based learning forms the basis of ambulatory rotations largely explaining the progress interns make during these rotations. In contrast, the classic learning model, which dominates the hospital internship rotations, is based on the teaching paradigm where learning is more passive [10, 11]. In competency-based learning, evaluations must be ongoing to ensure evaluation for learning rather than evaluation of learning [27]. Evaluations should prioritise continuous formative feedback to nurture intern progress. A summative approach can also be used to guide competency development [27]. Furthermore, self-evaluation increases the intern’s involvement and improves their perception of autonomy, control, competency, and personal efficiency [28].

Internship tutors monitor intern competency acquisition throughout their training. They also supervise the writing and presentation of complex cases and situations, which are additional more subjective and non-standardized evaluation tools, grouped into a portfolio. However, COVID led to pedagogical restructuring in Brest with tutor-intern meetings being reduced by half. Instead, reflective groups have been introduced for interns, often led by a supervisor and not the tutor. This could explain why participants questioned the legitimacy of tutors for monitoring competency acquisition during internships.

The final internship evaluations provide a more formal but friendly environment to assess the intern’s progress and achievements. Interns placed great importance on being listened to and having the ability to express themselves during evaluation sessions which is reflected in other literature [29]. The Exceler evaluation tool could facilitate a more constant and applied evaluation which possibly explains the positive comments on its exhaustiveness, despite this making it less feasible. It does not intend to replace the other evaluation methods, like complex situation reviews, but to complete them.

The intrinsic difficulties associated with Exceler noted during the study are consistent with the literature [17]. The novice level indicators often are not competency-based making their interpretation difficult. Participants also saw them as infantilising. The exhaustiveness of the table and the long formulation of certain indicators reduce feasibility and affect reflexivity [30]. Using the table in Excel® affects its readability making it harder to use. The table is intended for normative evaluation by setting benchmarks for learning in the curriculum [17]. It does not take into account the competency acquisition process as well as competencies that can be acquired elsewhere than in rotations. Therefore, it remains insufficient for certification on its own [15].

Participants also expressed the desire to adapt the evaluation tool to each rotation but this risks fragmenting learning and restricting the vision of real practice [31]. It could however be interesting to use Exceler for hospital internships since this may improve consistency between hospital and ambulatory rotations. Embedding Exceler in the portfolio would also lead to more regular meetings with the tutor who would have an additional tool for certifying competencies. Its use would also facilitate appropriation of competency-based learning model for interns and teachers [17, 32].

However, the introduction of a new evaluation table is often met with barriers. Despite these barriers, the University of Laval in Canada has successfully developed and implemented a criterion-referenced and computerised evaluation table called DBS-FM (Developmental Benchmarks Scale for Family Medicine). Its design is similar to Exceler and has the same objectives. However, the DBS-FM goes further in that it suggests how the intern is progressing compared to the level expected at the end of a rotation. It therefore proposes predefined “pedagogical prescriptions” meaning it could potentially go as far as suggesting whether or not an intern has successfully completed their rotation [33, 34]. The DBS-FM illustrates the potential offered by these types of tools and the likely growth in general medicine.

If Exceler was implemented, its use would have to be sufficiently explained to ensure that teachers and interns have a good command of it. Some indicators need to be reformulated to support interpretation standardisation. Furthermore, the motivation of both teachers and interns to use the table is essential [35]. This study revealed good acceptability with interns, but their opinion still needs to be confirmed after a practical use in real situations. Also, it would be interesting to collect the opinions of supervisors, tutors and more practice centers. These future studies could help to gain further understanding about potential barriers to implementation.

The main strength of this study was the use of the Dixit® game during the focus groups. Making the focus groups more like a game acted as an icebreaker and helped to instil a climate of trust. This also probably helped to reduce participant apprehension about being judged by others thus facilitating spontaneity and sincerity. The game encouraged everyone to justify and discuss their opinion and helped them to stay focused all along. Using this game enabled BL to run productive focus groups despite his lack of training and experience as an interviewer. The questions in the interview guide had been validated by the researchers and were open-ended. Therefore, the loss of data linked to differences in participant involvement, the researcher’s lack of experience and the absence of an observer was probably limited. BL’s lack of experience in the correct use of Exceler may have generated a lack of explanation which could have biased certain opinions. Data completeness, adherence to the COREQ checklist and analysis triangulation support the internal validity of the results.

## Conclusion

This study revealed the acceptability of the Exceler evaluation tool with general medicine interns. Exceler would improve evaluation quality, provide benchmarks for progress, and enable individualised evaluations. Furthermore, it would facilitate the appropriation of the competency-based learning model which is still insufficiently integrated. However, barriers still exist to it being used throughout the faculty. Analysing the opinions of tutors and supervisors could help to identify potential barriers and reveal solutions to facilitate its implementation and use.

### Electronic supplementary material

Below is the link to the electronic supplementary material.

Supplementary Material 1

Supplementary Material 2

## Data Availability

The datasets used and analysed during the current study are available from the corresponding author on reasonable request.

## Abbreviations

DBS-FM: Developmental Benchmarks Scale for Family Medicine
GP: General Practitioner
