# Supplementary material for: Acceptability and feasibility of an evaluation table to assess the competency of general medicine interns during ambulatory rotations in Brest
Source: BMC Med Educ. 2024 Jun 6;24:636. doi: 10.1186/s12909-024-05357-7 (PMC11157886; doi:10.1186/s12909-024-05357-7)
Supplement: Supplementary file 2 — Supplementary Material 2 [file 12909_2024_5357_MOESM2_ESM.docx]

Appendix 2: Interview guide

| **Opening Question** | **Follow-up questions** | **Relaunch points** |
| --- | --- | --- |
| 1. Which card best represents your last internship evaluation? | - Were there one or more interviews during the internship? If yes, at what times?  - Was there support for the assessment? If so, what did you think of it? | - Types of assessments encountered: formative / summative, criterion-referenced / normative  - Feelings about these evaluations, expectations  - Use of a support tool (evaluation table, etc.): usefulness, interest |
| *Presentation of the faculty evaluation table* | | |
| 2. Which card best represents your opinion about this evaluation table? | - Was it suitable for the evaluation of your internships?  - Is it precise enough?  - Is it subjective? Objective? | - Usefulness and positive / negative feedback |
| *Presentation of the Exceler evaluation table, explanation about its creation*  *(Quick review of GP competencies, explanation about evaluation table criteria)* | | |
| 3. Which card best represents your initial feeling about this evaluation table? | - What do you think of the support tool? And its use? | - Support  - Functioning  - Initial thoughts about this type of tool  - Discussion about formative / summative assessment methods  - Discussion about types of evaluation tables |
| *Distribution of different parts of the evaluation table to each participant by generic competency* | | |
| 4. Which card best represents the wording of the items, their sequence, their quantity? In other words, the quality of this evaluation grid? | - Are the items formulated in a sufficiently clear manner, are they understandable?  - Do you understand their sequence?  - Are there enough? Or on the contrary not enough? | - Content: quantity? Suitable? Clarity? (wording of items, quantity, sequence, etc.)  - Intrinsic strengths and weaknesses |
| *GP interns in Rennes use this evaluation table for self-assessment at the end of all their internships.*  *Limoges GP interns use this evaluation table:*   - *for self-assessment during all their internships, even hospital ones,* - *for their annual tutor evaluation and for their supervisor evaluation during each internship* - *at the end of the course to determine whether or not the intern has successfully completed their GP diploma.* | | |
| 5. If the decision about whether or not an intern had successfully completed their GP diploma was only based on this evaluation table, which card would best represent the state of mind of a Rennes/Limoges intern during an evaluation at the end of the internship? | - How could this evaluation table be **useful** to an intern / supervisor / tutor...?  - What **impact** would this evaluation table have on the interns / supervisors / tutor (motivation, involvement in the internship, source of stress, etc.)  - How would you use it? (self-assessment, supervisor assessment, in pairs during an interview?)  - How much **time** would you devote to completing it?  - When should it be used (beginning/middle/end)?  - What can a criterion-referenced evaluation table provide compared to other evaluation methods (GRIF, RSCA, etc.)? | - Use for interns, supervisors, tutors, teachers  - Motivation / constraint for interns / supervisors / tutors?  - Self / Hetero - evaluation?  - Expected benefits, motivation  - Negative consequences  - Regularity  - Formative and/or summative assessment  - Criterion-based or normative evaluation?  - Validating?  - Time spent  - Help with learning, gain in objectivity or, on the contrary, standardisation of learning? |
| 6. Which card best represents your overall impression of this evaluation table? | - Would you like it to be used?  - What should be improved?  - Is there a benefit to using such a tool? | - Student interest in the grid  - Possible improvements |

GP: General Practice

GRIF: Intern Reflective Groups

RSCA: Authentic Complex Situation Story
